# Use of Autologous Bone Graft with Bioactive Glass as a Bone Substitute in the Treatment of Large-Sized Bone Defects of the Femur and Tibia

**DOI:** 10.3390/jpm13121644

**Published:** 2023-11-24

**Authors:** Sebastian Findeisen, Niklas Gräfe, Melanie Schwilk, Thomas Ferbert, Lars Helbig, Patrick Haubruck, Gerhard Schmidmaier, Michael Tanner

**Affiliations:** Clinic for Trauma and Reconstructive Surgery, Center for Orthopedics, Trauma Surgery and Paraplegiology, Schlierbacher Landstraße 200a, University Hospital Heidelberg, 69118 Heidelberg, Germany; niklas.graefe@med.uni-heidelberg.de (N.G.); melaniesonja.schwilk@med.uni-heidelberg.de (M.S.); thomas.ferbert@med.uni-heidelberg.de (T.F.); lars.helbig@med.uni-heidelberg.de (L.H.); patrick.haubruck@sydney.edu.au (P.H.); gerhard.schmidmaier@med.uni-heidelberg.de (G.S.); m.tanner@corpus-mvz.de (M.T.)

**Keywords:** bioglass, S53P4, nonunion, segmental bone defect, Masquelet technique, bone healing, bone reconstruction

## Abstract

Background: Managing bone defects in non-union surgery remains challenging, especially in cases of large defects exceeding 5 cm in size. Historically, amputation and compound osteosynthesis with a remaining PMMA spacer have been viable and commonly used options. The risk of non-union after fractures varies between 2% and 30% and is dependent on various factors. Autologous bone grafts from the iliac crest are still considered the gold standard but are limited in availability, prompting consideration of artificial grafts. Objectives: The aims and objectives of the study are as follows: 1. To evaluate the radiological outcome of e.g., the consolidation and thus the stability of the bone (three out of four consolidated cortices/Lane-Sandhu-score of at least 3) by using S53P4-type bioactive glass (BaG) as a substitute material for large-sized bone defects in combination with autologous bone using the RIA technique. 2. To determine noticeable data-points as a base for future studies. Methods: In our clinic, 13 patients received bioactive glass (BaG) as a substitute in non-union therapy to promote osteoconductive aspects. BaG is a synthetic material composed of sodium, silicate, calcium, and phosphate. The primary endpoint of our study was to evaluate the radiological consolidation of bone after one and two years. To assess bone stabilization, we used a modified Lane-Sandhu score, considering only radiological criteria. A bone was considered stabilized if it achieved a minimum score of 3. For full consolidation (all four cortices consolidated), a minimum score of 4 was required. Each bone defect exceeded 5 cm in length, with an average size of 6.69 ± 1.92 cm. Results: The mean follow-up period for patients without final bone consolidation was 34.25 months, with a standard deviation of 14.57 months, a median of 32.00 months and a range of 33 months. In contrast, patients with a fully consolidated non-union had an average follow-up of 20.11 ± 15.69 months and a range of 45 months. Overall, the mean time from non-union surgery to consolidation for patients who achieved final union was 14.91 ± 6.70 months. After one year, six patients (46.2%) achieved complete bone consolidation according to the Lane-Sandhu score. Three patients (23.1%) displayed evident callus formation with expected stability, while three patients (23.1%) did not develop any callus, and one patient only formed a minimal callus with no expected stability. After two years, 9 out of 13 patients (69.2%) had a score of 4. The remaining four patients (30.8%) without expected stability either did not heal within two years or required a revision during that time. Conclusions: Bioactive glass (BaG) in combination with autologous bone (RIA) appears to be a suitable filler material for treating extensive non-unions of the femur and tibia. This approach seems to show non-inferiority to treatment with Tricalcium Phosphate (TCP). To ensure the success of this treatment, it is crucial to validate the procedure through a randomized controlled trial (RCT) with a control group using TCP, which would provide higher statistical power and more reliable results.

## 1. Introduction

The management of bone defects in non-union surgery remains challenging. In the literature, the risk of developing a non-union after fracture is approximately 2% to 30% over all locations and is dependent on multiple factors such as sex, age, smoking status, diabetes and vascular status. Anatomically, the highest percentage of non-unions affect the tibia and the femur, which may impede the grade of mobility of the patient, and thereby their quality of life itself [1,2].

In the past, amputation, compound osteosynthesis with a remaining PMMA spacer, and distraction techniques or vascularized bone grafts have been the most viable and common treatment options. In particular, large-sized defects, generally classified as defects over 5 cm and with a substantial loss of bone volume, have been one of the hardest problems to treat [3,4]. The choice of treatment has a significant impact on the patient with regard to the expected time of therapy. If not handled correctly, the outcome can rapidly become a burden. Careful planning is paramount in the successful treatment of these entities.

Autologous bone graft remains the gold standard for treatment of bone defects, but its availability is limited therefore, only a restricted number of surgeries can be performed. Artificial bone grafts may be applied in addition to or even replace ABG in certain circumstances, because of their reasonably unlimited availability and promising characteristics. In recent years, besides autologous bone multiple bone substitute materials have been offered for treatment.

Patients in the University Hospital Heidelberg are treated via a standardized protocol, specifically in accordance with the diamond concept. This concept was originally introduced by Giannoudis et al. in 2007, initially including four criteria: mechanical stability, osteogenic cells, osteoinductive mediators and osteoconductive matrix e.g., scaffold [5,6]. In 2008 Giannoudis P, Einhorn M, and Schmidmaier G expanded the diamond concept five factors, adding vascularization [7,8].

## 2. Materials and Methods

### 2.1. Study Design

**Figure 1:**
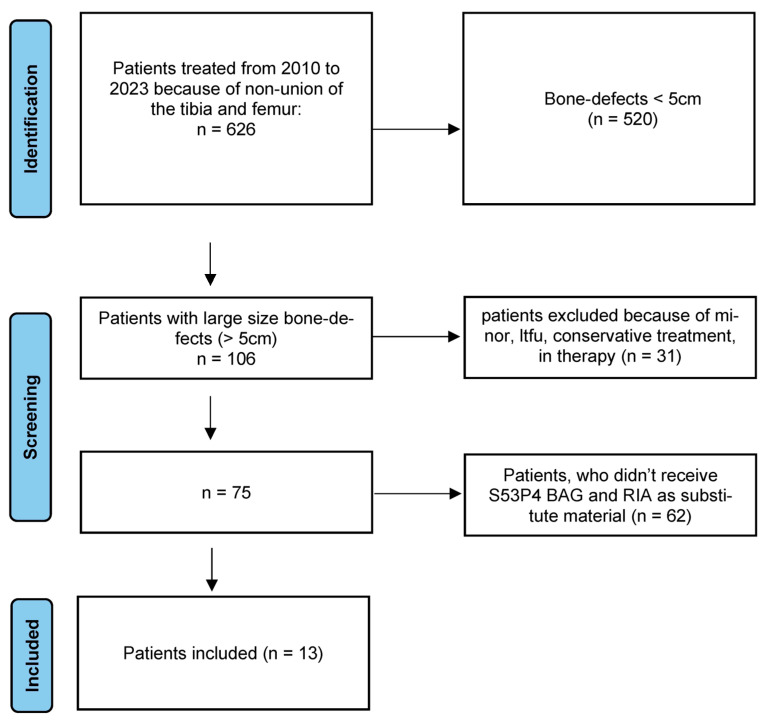
Screening process.

**Figure 2:**
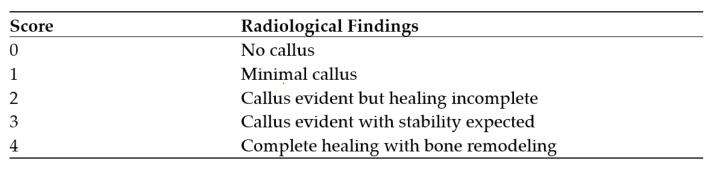
Modified Lane-Sandhu-score.

This study was designed as a single-center retrospective descriptive database analysis. Data were based on the clinical database of treated non-unions in the University Hospital Heidelberg, Department of Trauma and Reconstructive Surgery. The local ethics committee granted ethical approval (S-262/2017) and the study was conducted in accordance with the Declaration of Helsinki. From 1 January 2010 to 31 December 2022 626 patients with non-unions of the tibia or femur were treated in our hospital, of which 106 patients had large-sized defects (>5 cm). Amongst the latter, 31 patients had to be excluded, because of loss to follow-up. Ultimately, 13 of the remaining 75 patients received BaG as a bone substitute in combination with an autologous bone graft in RIA technique. During the period from 2010 to 2017, all patients with segmental defects using the Masquelet technique were managed with autologous iliac crest bone graft or bone graft harvested using the Reamer-Irrigator-Aspirator (RIA) technique, with Tricalcium Phos-phate (TCP) as the bone substitute. In total, there were n = 62 cases. Starting in 2018, we adopted bioactive glass as the standard bone substitute instead of TCP in all cases in the surgical treatment of large-sized non-unions. Accordingly, since 2018, all patients with a large size defect have received BaG as a bone substitute (n = 13). The whole screening process was shown in Figure 1. The primary endpoint was radiological consolidation after one and two years. Secondary endpoints were stabilization after one und two years and occurrence of complications or infections. Each patient was monitored for risk factors such as smoking, adipositas, comorbidities, medications, type of fracture and NUSS-score. For assessment of osseous stabilization, a modified Lane-Sandhu-score (Figure 2) was used, including only radiological criteria. A bone was considered stabilized if a minimum score of 3 was achieved. For consolidation (4 out of 4 cortices consolidated) a minimum score of 4 had to be achieved [9,10].

**Figure 3:**
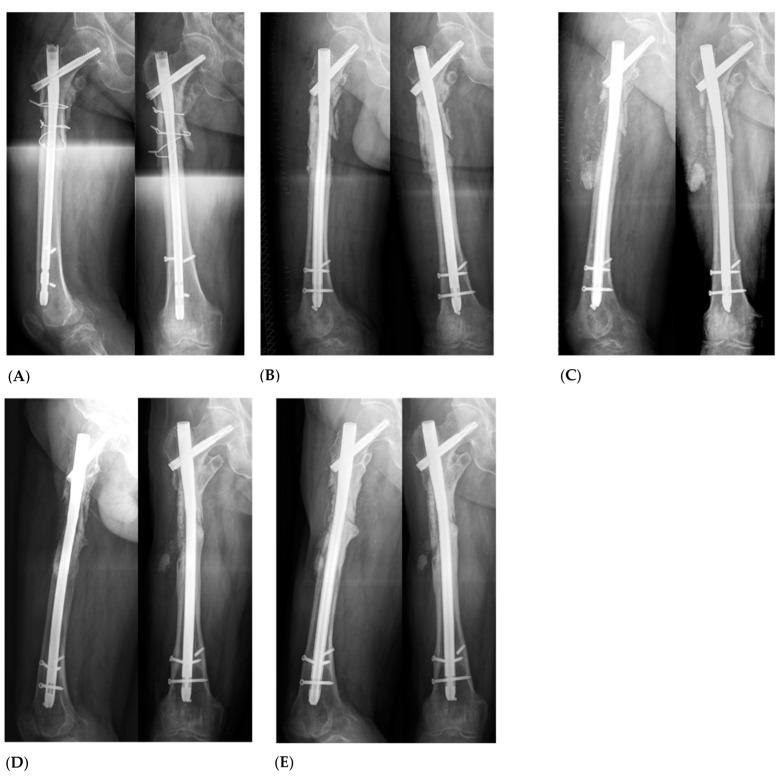
Case of a 55-year-old patient with atrophic nonunion following nail fixation of the proximal femur after subtrochanteric femur fracture (**A**). After performing Masquelet Step I with debridement of the atrophic bone, a significant bone defect exceeding 5 cm in size was observed (**B**). To address it, BaG was used as a bone substitute in combination with autologous bone using the RIA (Reamer Irrigator Aspirator) technique (**C**). At the 1-year follow-up (**D**), X-rays revealed complete bone consolidation, and ongoing bone remodeling was observed after 2 years (extended follow-up) (**E**).

In order to address vascularization, all patients were treated with a two-staged induced membrane technique, first described by Masquelet A. in 2000 [11]. This procedure involves at least two operations. Figure 3 exemplifies the radiological follow-up of a study patient with a large size defect of the proximal femur (Figure 3).

### 2.2. S53P4-Type Bioactive Glass (BaG)

In total, 13 patients received autologous bone via RIA in combination with BaG as a substitute during the second stage of the induced membrane technique. BaG is a synthetic material consisting of sodium, silicate, calcium and phosphate. It showed satisfying results in improving the rate of bone union because of its osteoconductive, antimicrobial and osteostimulative effects. The release of calcium ions results in the formation of hydroxyapatite. [12,13,14,15,16] The use of BaG has not shown any relevant side effects [17,18,19]. Therefore, BaG seems like a promising option for the treatment of bone non-unions.

### 2.3. Statistical Analysis

Statistical Program for Social Science (SPSS) Version 29.0 was used to analyze the data. Quantitative data were expressed as mean with the standard deviation (±for SD) and the median was added for quantitative data of time. Qualitative data were described by using the absolute count and the relative rate (in %). Due to the low patient count, no tests for significance are shown.

Summarized aims and objectives of the study are:

1. To evaluate the radiological outcome of, e.g., consolidation and thus the stability of the bone (3 out of 4 consolidated cortices/at least Lane-Sandhu-score of 3) by using S53P4-type bioactive glass (BaG) as substitute material for large-sized bone defects.
2. To determine noticeable data points as a base for future studies.

## 3. Results

### 3.1. Patient Characteristics

The mean age of the patients was 55.30 years with a minimum age of 35 and a maximum of 87 years. The majority was male with 9 out of 13 patients (69.2%) and 4 were female (30.8%). Every bone defect was greater than 5cm in length, with a mean of 6.69 ± 1.92 cm. In total, 76.9% of the non-unions were localized in the tibia, while 23.1% were in the femur. [16] With regard to risk factors, the smoking status was relatively balanced between smokers (46.2%) and non-smokers (46.2%). One patient was a former smoker (7.7%). The ASA-score averaged at 1.92 ± 0.28 points and 15.4% (2 in total) of patients had diabetes mellitus. Amongst the study cohort, 15.4% (2 in total) had Grade I adipositas (BMI between 30 and 34.9) and one patient (7.7%) had grade III adipositas, whereas 76.9% were of regular weight. The average BMI was 28.27 ± 5.11 (Table 1).

### 3.2. Follow Up

**Figure 4:**
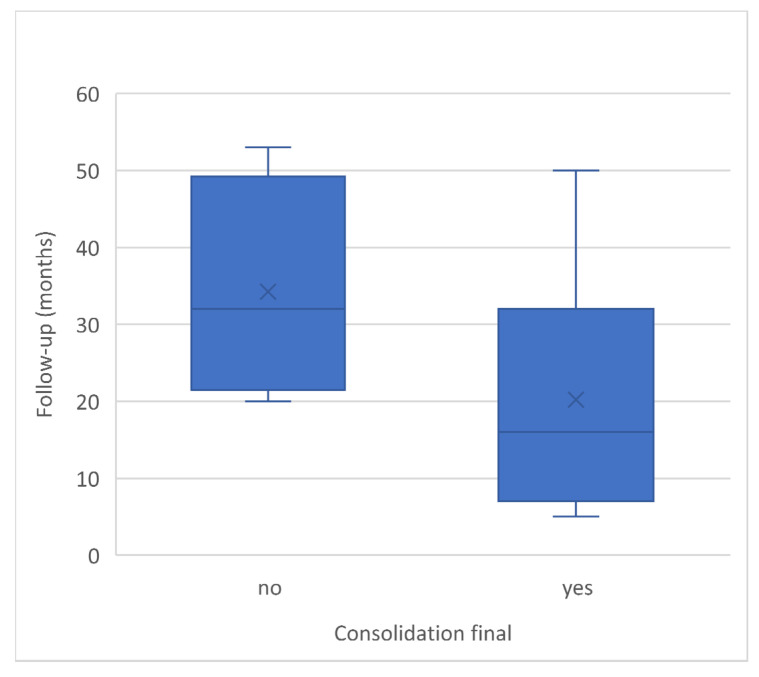
Consolidation final.

Patients who did not achieve final consolidation had an average follow-up duration of 34.25 months, with a standard deviation of 14.57 months. The median follow-up time was 32.00 months, with a minimum of 20 months and a range of 33 months. On the other hand, patients with consolidated non-unions had an average follow-up period of 20.11 months, with a standard deviation of 15.69 months. The minimum follow-up time was 5 months, and the range extended to 45 months, with a median follow-up duration of 16.00 months. (Figure 4). In total the mean time from non-union surgery to-consolidation for patients with final union was 14.91 ± 6.70 months and a median of 14.08 months. The time from trauma to consolidation showed a mean of 36.56 months ±13.60 and a median of 34.00 months (Table 2). The shortest duration between trauma and the initial non-union surgery at our clinic was 9 months, while the longest was 25 months. The average time for the entire group was 16.50 months with a standard deviation of 5.30 months, within a range of 16 months.

After one year, six patients (46.2%) achieved full bone consolidation according to the Lane-Sandhu-score. Three patients (23.1%) had an evident callus, and therefore a score of 3. Furthermore, three patients (23.1%) did not develop callus and one patient only formed a minimal callus with no stability expected.

After two years, 9 out of 13 (69.2%) patients had a score of 4. All three patients with a Lane-Sandhu-score of 3 one year before achieved complete bone healing at two years. The three patients (23.1%) without expected stability did not heal in two years or had been revised in the meantime. One patient was amputated and two patients underwent revision surgery (one after 9 months [P1] and the other after 20 months [P2]). Out of the latter two patients, one (50.0%) consolidated after 1.5 years [P1]. Patients with surgical revision were considered final non-union (Table 3). The first patient [P1] had three surgeries after the initial bone reconstruction attempt for a tibial diaphysis defect, which healed after addressing the fistula, sequestrectomy, and using BaG Granules with autologous bone graft. The second patient [P2] experienced implant failure in the distal femur. Revision took place 20 months after the initial Masquelet Step II, involving plate replacement and BaG/TCP substitution. After 14 months, the bone defect semi-consolidated, achieving a Lane-Sandhu-score of 3.

The overall healing rate for non-unions of the tibia and femur was 46.2% (6/13) after one year and 69.2% (9/13) after two years. The monitored risk factors showed that in the group of smokers, four patients (66.6%) achieved a one-year-consolidation and after two years 83.3% (5/6) accomplished a consolidation of their non-union. One smoker did not consolidate. In the non-smoker group healing took place in one patient (16.7%) after one year and in three patients (50.0%) after two years. Healing took place in one case for a patient, who was a former smoker after one year as well as after two years. Two patients were screened for consolidation and had diabetes mellitus. One out of two (50.0%) achieved full consolidation after one and after two years. The rate of consolidation in patients with adipositas was 0% after one year and 33% after two years. Almost all patients (10/13) had a BMI under 30. They had a consolidation rate of 60.0% (6 out of 10) after one year and 80.0% (8 out of 10) after two years.

In 38.5% of cases (5/13), a positive bacterial culture was obtained after performing Masquelet Step I. These included two cases of Staphylococcus aureus, and one case of Staphylococcus capitis, epidermidis, lugdunensis, and saccharolyticus. In these instances, a repeat radical debridement with spacer exchange was performed until no bacteria were detectable in the tissue samples, allowing us to confirm the absence of infection. In this group, 20.0% (1/5) achieved consolidation after one year, and 40.0% (2/5) achieved consolidation after two years.

Complications during the non-union therapy were documented in 46.2% (6 out of 13) of patients. Complications included delayed wound healing (2/6), implant failure (1/6), bone fistulas (1/6), unplanned revision surgery due to postoperative malrotation of the tibia (1/6) and amputation (1/6). Out of those 50% (3/6) with complications consolidated after one year and 66.6% patients after two years (4/6). Hence, 75.0% (3/4) of the group with no final bony consolidation had complications during their non-union therapy. The initial fractures of the monitored patient-collective were classified as “closed” (69.2%; 9/13), “open 1°” (7.7%; 1/13), “open 2°” (15.4%; 2/13) and “open 3°” (15.4%; 2/13) according to Tscherne and Oestern [20]. Full consolidation took place in 55.6% (5 out 9) of closed factures after one year and in 88.9% after two years. The patient with an open 1° fracture did not consolidate, as well as the two patients with open 3° fractures. Two patients had open 2° fractures and both (100.0%) fully consolidated after one year (Table 4).

Comparing the age between final non-unions and consolidation after two years showed that the mean age of patients without consolidation after one year was 56.14 years with a standard deviation of 10.85 years. The mean age of patients with consolidation after one year was 54.33 years with a standard deviation of 18.11 years. After 2 years the mean age of patients without healing was 55.00 ± 11.69 years and that of patients with a full consolidation was 55.44 ± 15.60 years. BMI showed a mean score of 29.89 ± 5.62 for patients without consolidation after one year and 31.18 ± 6,01 after two years. BMI for patients who healed was 26.01 ± 3.65 after one year and 26.82 ± 4.27 after two years. The Non-Union Scoring System [21] was screened and showed a mean score of 47.14 ± 9.99 points for patients without consolidation and 45.00 ± 8.07 points for patients with consolidation after one year. This mean score was higher for the final non- consolidation group, with 50.50 ± 11.47 points and lower for the final-consolidation group with 44.22 ± 7.38 points (Table 5).

## 4. Discussion

The goal of our study was to validate our treatment of tibial and femoral large-sized non-unions according to the diamond concept, using BaG as substitute material in combination with autologous bone in RIA technique. Since the diamond concept was implemented in 2010 in our department, the same conditions have been applied for all patients. From this time forward, many patients with non-unions of the lower limb have been treated accordingly, although non-unions with large size bone defects are still rare. With a consolidation rate of 69.2% after two years, the outcome is comparable to other studies performed [22].

It is difficult to place our consolidation rate of 69.2% in context with other studies, due to a lack of evidence in the literature. Konda et al. recently published a paper about the outcome of the IMT for bones with a diaphyseal large segmental bone loss. Some of the few studies that show a defect size similar to ours were 8.85 cm (Konda et al.)—6.60 cm in (Findeisen et al.) [1,23]. They achieved an impressing consolidation rate of 88.2% by using different types of substitute materials, e.g., cancellous bone chip allograft used as a volume expander in 70.6% and also RIA in 70.6%. It is difficult to compare our study to these results, because of the diverse characteristics. Konda et al. included bones from the upper and lower extremities. They also used many different bone graft sources (four and above in 41.6% of the cases) and a revision with a repeat bone grafting was not considered as a failure, in contrast to our study. The results showed that IMT is a good viable option to treat patients with a segmental large bone loss, but cannot be compared in aspects of the better substitute material [23].

In 2017 Moghaddam et al. published an article about non-unions of the femoral shaft with a mean bone-defect size of 4.8 ± 3.9 cm. This study showed a consolidation rate of 64%. Including the bone size as a risk factor for final non-unions our findings can be acknowledged as a satisfying final result. Bone healing can take place even if there is no bone bridge left, which was not achieved in these studies [22]. A weakness of this study was its setting with retrospective inclusion and no randomization. The latter was not possible because of the missing control-group due to the small patient collective. Large-sized bone defects are a rarity, even within the realm of non-unions. Adding a control-group with the previous prevailing TCP + RIA as substitute materials would have resulted in a much smaller comparison group. The expressive power can be increased by planning a multicenter prospective randomized study with a control and a comparison group.

Similarly, the results of our study must be contextualized with other approaches, apart from the one following the Masquelet technique, for treating large-sized defects, such as those involving callus distraction. For instance, Sigmund IK et al., in their prospective multicenter study from 2020, described a consolidation rate of 100% in both study groups for infected segmental bone defects. They included 47 patients with defects > 2 cm on the tibia who were treated using callus distraction with an Ilizarov fixator (bifocal acute shortening and relengthening) or bone transport. However, despite the high consolidation rate, the number of unplanned revision surgeries was relatively high (38%). The authors also confirmed the known issues with the docking site, albeit significantly lower in the ASR group. Nonetheless, it is important to differentiate that this study examined large size defects of a short duration with an average of three previous surgeries. Therefore, randomized controlled trials (RCTs) are needed to assess the effectiveness of bioglass as a bone substitute and to enable comparisons with other methods [24].

Final non-union follow-up was on average eleven months longer, indicating that the higher consolidation rate did not depend on longer follow-up. The minimum follow-up period for patients with definitive non-union was 24 months. Patients who consolidated earlier did not need to be followed up for the full 24 months, which explains the mean follow-up time of 20.11 months. With an average healing time of 14.91 months, the time is slightly longer than in other studies. This may be due to the fact that the defect size was larger in comparison [22].

Regarding the risk factors, they seem to be in line with what is known for the outcome of non-union of the long bone so far. Risk factors such as adipositas and infections during non-union therapy appeared to have a negative impact on healing time and final healing. This was also described previously in the scientific literature [25,26,27]. By looking at the BMI between the consolidation group and the non-consolidation group, it can be seen that the BMI was higher by at least 4 points both after one and two years for the non-consolidation group. It was shown previously, that an increased BMI has a negative impact on the consolidation rate [28]. Other potential risk factors such as smoking, diabetes or age did not seem to have an impact on the primary outcome, even though it has been shown that these can affect the occurrence and the result of non-union [29,30,31]. This finding is most probably due to the low number of cases in this study. By extension, it cannot be proven that infections or adipositas have a significant impact either. The complications appear to also have a negative impact on healing, but because of the wide range of different complications it is hard to make a final statement. It is more likely that patients with worse health conditions had a higher chance of developing a complication and, for the same reason, a worse outcome with respect to the healing of non-unions.

For the radiological evaluation of bone consolidation during follow-up, we employed the Lane Sandhu score. This score considers not only the healing of the bone’s outer layer (cortical healing) but also takes into consideration the integrity of the osteosynthesis, which is particularly crucial in cases involving large defects. After one year, a noteworthy proportion of patients achieved a Lane Sandhu score of 3, indicating that the combination of the implant and the fully healed bone creates a stable structure, eliminating the need for any further interventions.

When considering the consolidation rate of cases with a positive intraoperative bac-terial detection (5/13), it is significantly worse than that of the overall cohort (1/5 after one year, 2/5 after two years). This reinforces that patients with open fractures and persistent low-grade infections have a poorer outcome. A similar conclusion was drawn when using BaG stand alone in cases of infected non-unions, where BaG exhibited a high rate of rein-fection as well [32]. In a retrospective single-center study, Steinhausen et al. reported on the treatment of 50 patients with chronic osteomyelitis and infected non-unions, using BaG (46 cases) and in combination with autologous bone (4 cases). They observed a higher reinfection rate in the BaG-treated group, with Staphylococcus aureus being the most common pathogen identified [32]. In the mentioned study, BaG mono without autologous cancellous bone was predominantly used in the majority of cases, making the results not directly applicable to ours. Further studies with larger patient cohorts are needed to inves-tigate the potential increased infection rate associated with BaG. However, it is important to note that the limited number of cases in our cohort (n = 13) prevents us from drawing a definitive conclusion that the combination of BaG with autologous bone in cases of infec-tion represents a suboptimal treatment option.

By means of the NUSS-score, the average physical condition can be assessed. The group that did not achieve a final union had a 5-point higher mean score. This underpins the statement that this group had a worse physical state. The NUSS-score includes 15 different items, grouped in three macrodomains, e.g., smoking, clinical infection status or diabetes mellitus [21]. Gaddi et al. assumed that the NUSS-score may underestimate the medical therapy required [33]. Our patients were classified as NUSS 2 and 3, but for similar reasons, the same medical protocol was performed for both groups.

### Limitations

This study has several limitations. First, over a period of more than 4 years, only 13 patients with large-sized defects were included, despite our clinic specializing in the treatment of non-unions. However, since there are currently no comparable studies investigating the use of bioactive glass in the treatment of large sizes bone defects, we still consider the results relevant. Due to the national and international patient pool, our study had a relatively high loss to follow-up rate, as patients, especially when symptom-free, sought further treatment closer to their homes. Additionally, the retrospective study design represents a significant limitation. To ensure the success of this kind of treatment it is essential to validate the procedure by means of an RCT with a control group using, e.g., TCP for a higher power. Furthermore, consideration should be given to a multicenter study design to further increase the test power.

## 5. Conclusions

BaG in combination with autologous bone (RIA) appears to be a suitable filler material for extensive non-unions of the femur and tibia and, according to this study, seems to show non-inferiority to the treatment with TCP.

Moreover, it seems that revision-surgery should not be performed too early when there is no implant failure but complete healing has not yet occurred. This is corroborated by the findings in the Lane-Sandhu score, where all of our patients with a score of 3 after one year, converted to a score of 4 after two years. Four of our patients (26.7%) had no complete healing with bone remodeling after one year, but from a clinical point of view the non-union was categorized as stable. For each of the four patients bone remodeling even took place in the second year. As shown in previous publications, it seems that the time span of most studies is too short with a follow-up of under 2 years. It should be stressed, that with every additional revision surgery the consolidation rate decreases. [3,34,35] To evaluate this, a long-term study should be performed.

## Figures and Tables

**Table 1:** Patient characteristics.

		Bone Substitute			
		S53P4			
		Count	n%	Mean	Standard Deviation
sex	female	4	30.8%		
	male	9	69.2%		
	age			55.30	14.01
bone defect before surgery (cm)				6.69	1.92
location	femur	3	23.1%		
	tibia	10	76.9%		
smoking	non smoker	6	46.2%		
	previous smoker	1	7.7%		
	smoker	6	46.2%		
	ASA			1.92	0.28
diabetes mellitus	no	11	84.6%		
	yes	2	15.4%		
adipositas	adipositas	3	23.1%		
	no adipositas	10	76.9%		
	BMI			28.27	5.11

**Table 2:** Final consolidation.

	Final Consolidation					
	No			Yes		
	Mean	Median	Standard Deviation	Mean	Median	Standard Deviation
follow-up (months)	34.25	32.00	14.57	20.11	16.00	15.69
non-union to consolidation				14.91	14.08	6.70
trauma to consolidation				36.56	34.00	13.60

**Table 3:** Lane-Sandhu-score of the cohort after one and two years.

		Bone Substitute	
		S53P4	
		Count	n%
Lane-Sandhu-score after 1 year	no callus	3	23.1%
	minimal callus	1	7.7%
	callus evident but healing incomplete	0	0.0%
	callus evident with stability expected	3	23.1%
	complete healing with bone remodeling	6	46.2%
Lane-Sandhu-score after 2 years	no callus	3	23.1%
	minimal callus	1	7.7%
	callus evident but healing incomplete	0	0.0%
	callus evident with stability expected	0	0.0%
	complete healing with bone remodeling	9	69.2%

**Table 4:** Correlation between pre-existing conditions (comorbidities) and consolidation rate.

.		Consolidation after 1 Year				Consolidation after 2 Years			
		No		Yes		No		Yes	
		Count	n%	Count	n%	Count	n%	Count	n%
total		7	53.8%	6	46.2%	4	30.8%	9	69.2%
smoking	non-smoker	5	83.3%	1	16.7%	3	50.0%	3	50.0%
	previous smoker	0	0.0%	1	100.0%	0	0.0%	1	100.0%
	smoker	2	33.3%	4	66.6%	1	16,7%	5	83.3%
diabetes mellitus	no	6	54.5%	5	45.5%	3	27.3%	8	72.7%
	yes	1	50.0%	1	50.0%	1	50.0%	1	50.0%
adipositas	adipositas	3	100.0%	0	0.0%	2	66.7%	1	33.3%
	no adipositas	4	40.0%	6	60.0%	2	20.0%	8	80.0%
positive bacterial culture	no	3	37.5%	5	62.5%	1	12.5%	7	87.5%
	yes	4	80.0%	1	20.0%	3	60.0%	2	40.0%
complication by nonunion treatment	no	3	42.9%	4	57.1%	1	14.3%	6	85.7%
	yes	4	33.3%	2	66.7%	3	50.0%	3	50.0%
type of fracture	closed	4	44,4%	5	55.6%	1	11.1%	8	88.9%
	open 1°	1	100.0%	0	0.0%	1	100.0%	0	0.0%
	open 2°	0	0.0%	2	100.0%	0	0.0%	2	100.0%
	open 3°	2	100.0%	0	0.0%	2	100.0%	0	0.0%

**Table 5:** Consolidation rate after one and two years.

	Consolidation after 1 Year				Consolidation after 2 Years			
	No		Yes		No		Yes	
	Mean	Standard Deviation	Mean	Standard Deviation	Mean	Standard Deviation	Mean	Standard Deviation
age	56.14	10.85	54.33	18.11	55.00	11.69	55.44	15.60
BMI	29.89	5.62	26.01	3.65	31.18	6.01	26.82	4.27
Non-Union-Scoring-System	47.14	9.99	45.00	8.07	50.50	11.47	44.22	7.38

## Data Availability

Data was collected from our local non-union database of our Institution.

## References

[1] Findeisen S., Schwilk M., Haubruck P., Ferbert T., Helbig L., Miska M., Schmidmaier G., Tanner M.C. (2023). Matched-Pair Analysis: Large-Sized Defects in Surgery of Lower Limb Nonunions. J. Clin. Med..

[2] Mills L.A., Aitken S.A., Simpson A. (2017). The risk of non-union per fracture: Current myths and revised figures from a population of over 4 million adults. Acta Ortho.

[3] Weinberg H., Roth V.G., Robin G.C., Floman Y. (1979). Early Fibular Bypass Procedures (Tibiofibular Synostosis) for Massive Bone Loss in War Injuries. J. Trauma: Inj. Infect. Crit. Care.

[4] Metsemakers W., Kuehl R., Moriarty T., Richards R., Verhofstad M., Borens O., Kates S., Morgenstern M. (2018). Infection after fracture fixation: Current surgical and microbiological concepts. Injury.

[5] Giannoudis P.V., Einhorn T.A., Marsh D. (2007). Fracture healing: The diamond concept. Injury.

[6] Andrzejowski P., Giannoudis P.V. (2019). The ‘diamond concept’ for long bone non-union management. J. Ortho Traumatol..

[7] Miska M., Schmidmaier G. (2020). Diamond concept for treatment of nonunions and bone defects. Unfallchirurg.

[8] Giannoudis P.V., Einhorn T.A., Schmidmaier G., Marsh D. (2008). The diamond concept--open questions. Injury.

[9] Lane J.M., Sandhu H.S. (1987). Current approaches to experimental bone grafting. Orthop. Clin. N. Am..

[10] Schnetzke M., Morbitzer C., Aytac S., Erhardt M., Frank C., Muenzberg M., Studier-Fischer S., Helbig L., Suda A.J., Gruetzner P.-A. (2015). Additional bone graft accelerates healing of clavicle non-unions and improves long-term results after 8.9 years: A retrospective study. J. Orthop. Surg. Res..

[11] Masquelet A.-C., Fitoussi F., Begue T., Muller G.P. (2000). Reconstruction of the long bones by the induced membrane and spongy autograft. Ann. Chir. Plast. Esthet..

[12] Aurégan J.-C., Villain B., Glombitza M., Blokhuis T., Heinänen M., Bégué T. (2022). Utilisation of bioactive glass S53P4 inside an induced membrane for severe bone defect with high risk of infection: A multi-center preliminary experience. Injury.

[13] Björkenheim R., Jämsen E., Eriksson E., Uppstu P., Aalto-Setälä L., Hupa L., Eklund K., Ainola M., Lindfors N., Pajarinen J. (2021). Sintered S53P4 bioactive glass scaffolds have anti-inflammatory properties and stimulate osteogenesis in vitro. Eur. Cells Mater..

[14] Eriksson E., Björkenheim R., Strömberg G., Ainola M., Uppstu P., Aalto-Setälä L., Leino V.-M., Hupa L., Pajarinen J., Lindfors N.C. (2021). S53P4 bioactive glass scaffolds induce BMP expression and integrative bone formation in a critical-sized diaphysis defect treated with a single-staged induced membrane technique. Acta Biomater..

[15] Freischmidt H., Armbruster J., Rothhaas C., Titze N., Guehring T., Nurjadi D., Sonntag R., Schmidmaier G., Grützner P.A., Helbig L. (2022). Treatment of Infection-Related Non-Unions with Bioactive Glass-A Promising Approach or Just Another Method of Dead Space Management?. Materials.

[16] Tanner M.C., Heller R., Westhauser F., Miska M., Ferbert T., Fischer C., Gantz S., Schmidmaier G., Haubruck P. (2018). Evaluation of the clinical effectiveness of bioactive glass (S53P4) in the treatment of non-unions of the tibia and femur: Study protocol of a randomized controlled non-inferiority trial. Trials.

[17] Geurts J., van Vugt T., Thijssen E., Arts J.J. (2019). Cost-Effectiveness Study of One-Stage Treatment of Chronic Osteomyelitis with Bioactive Glass S53P4. Materials.

[18] Drago L., Romanò D., De Vecchi E., Vassena C., Logoluso N., Mattina R., Romanò C.L. (2013). Bioactive glass BAG-S53P4 for the adjunctive treatment of chronic osteomyelitis of the long bones: An in vitro and prospective clinical study. BMC Infect. Dis..

[19] Van Gestel N.A., Geurts J., Hulsen D.J., van Rietbergen B., Hofmann S., Arts J.J. (2015). Clinical Applications of S53P4 Bioactive Glass in Bone Healing and Osteomyelitic Treatment: A Literature Review. Biomed. Res. Int..

[20] Tscherne H., Oestern H.J. (1982). A new classification of soft-tissue damage in open and closed fractures (author’s transl). Unfallheilkunde.

[21] Calori G.M., Phillips M., Jeetle S., Tagliabue L., Giannoudis P.V. (2008). Classification of non-union: Need for a new scoring system?. Injury.

[22] Moghaddam A., Thaler B., Bruckner T., Tanner M., Schmidmaier G. (2017). Treatment of atrophic femoral non-unions according to the diamond concept: Results of one- and two-step surgical procedure. J. Orthop..

[23] Konda S.R., Boadi B.I., Leucht P., Ganta A., Egol K.A. (2023). Surgical repair of large segmental bone loss with the induced membrane technique: Patient reported outcomes are comparable to nonunions without bone loss. Eur. J. Orthop. Surg. Traumatol..

[24] Sigmund I.K., Ferguson J., Govaert G.A.M., Stubbs D., McNally M.A. (2020). Comparison of Ilizarov Bifocal, Acute Shortening and Relengthening with Bone Transport in the Treatment of Infected, Segmental Defects of the Tibia. J. Clin. Med..

[25] Lu Y., Lai C., Lai P., Yu Y. (2023). Induced Membrane Technique for the Management of Segmental Femoral Defects: A Systematic Review and Meta-Analysis of Individual Participant Data. Orthop. Surg..

[26] Issace S.J.J., Singh R.S.J., Sisubalasingam N., Tokgöz M.A., Jaiman A., Rampal S. (2023). Does obesity affect diaphyseal femoral fracture healing treated with intramedullary locking nail?. Jt. Dis. Relat. Surg..

[27] Morelli I., Drago L., George D.A., Gallazzi E., Scarponi S., Romanò C.L. (2016). Masquelet technique: Myth or reality? A systematic review and meta-analysis. Injury.

[28] Tanner M.C., Heller R.A., Grimm A., Zimmermann S., Pilz M., Jurytko L., Miska M., Helbig L., Schmidmaier G., Haubruck P. (2021). The Influence of an Occult Infection on the Outcome of Autologous Bone Grafting During Surgical Bone Reconstruction: A Large Single-Center Case-Control Study. J. Inflamm. Res..

[29] Pearson R.G., E Clement R.G., Edwards K.L., E Scammell B. (2016). Do smokers have greater risk of delayed and non-union after fracture, osteotomy and arthrodesis? A systematic review with meta-analysis. BMJ Open.

[30] Tian R., Zheng F., Zhao W., Zhang Y., Yuan J., Zhang B., Li L. (2020). Prevalence and influencing factors of nonunion in patients with tibial fracture: Systematic review and meta-analysis. J. Orthop. Surg. Res..

[31] Moghaddam A., Zimmermann G., Hammer K., Bruckner T., Grützner P.A., von Recum J. (2011). Cigarette smoking influences the clinical and occupational outcome of patients with tibial shaft fractures. Injury.

[32] Malat A., Glombitza M., Dahmen J., PM H., Steinhausen E. (2021). Bioactive glass S53P4 vs. autologous bone graft for filling defects in patients with chronic osteomyelitis and infected non-unions—A single center experience. J. Bone Joint. Infect..

[33] Gaddi D., Gatti S.D., Piatti M., Poli A., De Rosa L., Riganti A., Zatti G., Bigoni M., Turati M. (2023). Non-Union Scoring System (NUSS): Is It Enough in Clinical Practice?. Indian J. Orthop..

[34] Benz D., Tarrant S.M., Balogh Z.J. (2020). Proximal femur fracture non-union with or without implant failure: A revision technique with clinical outcomes. Injury.

[35] Luo J., Bo F., Wang J., Wu Y., Ma Y., Yin Q., Liu Y. (2022). Application of the induced membrane technique of tibia using extracorporeal vs. intracorporeal formation of a cement spacer: A retrospective study. BMC Musculoskelet. Disord..

